# Healing of Large Endodontic Lesions Using Long-Term Application of a New Combination of Triple Antibiotics: A Series of Cases

**DOI:** 10.1155/2023/6889019

**Published:** 2023-04-05

**Authors:** Saeed Asgary, Ardavan Parhizkar

**Affiliations:** Iranian Centre for Endodontic Research, Research Institute for Dental Sciences, Shahid Beheshti University of Medical Sciences, Tehran 198396-3113, Iran

## Abstract

Apical periodontitis (AP) is defined as an inflammatory and destructive reaction of periapical tissues and a consequence of bacterial invasion to the dental pulp and root canal system. To avoid possible complications and undesirable repercussions of the surgical treatment of large AP, more conservative non-surgical approaches are endorsed. In the presented hopeless cases, a newly introduced modified combination of triple antibiotics, that is Penicillin G, metronidazole, and ciprofloxacin (PMC), was used as a long-term intracanal medication in the non-surgical endodontic retreatments of large AP. In the course of 10-month to 3-year follow-up, the large periapical lesions completely disappeared, and thorough bone healing was evident. Additionally, complete functionality of the involved teeth as well as other favourable treatment outcomes of the addressed cases showed that the long-term use of PMC, as a modified combination of antibiotics, in non-surgical endodontic retreatments may successfully resolve large AP. However, further investigations and well-designed controlled clinical trials are recommended.

## 1. Introduction

Apical periodontitis (AP) is described as the inflammation and destruction of periapical tissues and a response to damages to the dental pulp and root canal space, for example, infections of the radicular area, injuries following endodontic treatments, and consequences of root canal filling materials [1]. AP is addressed as complex pathology with inflammatory/anti-inflammatory molecules/mediators and their interactions, which can affect the condition, progression, and enlargement of AP. It seems that in the formation of AP, oral microbiota infects the pulpal tissue and causes degeneration of the dental pulp. Consecutively, microorganisms advance in the root canal system and invade the periapical region. However, the host defends itself through different mechanisms, various types of cells, and defence molecules, destroying periapical tissues resulting in AP [2].

One of the main objectives of endodontic treatment is the removal of etiological factors; therefore, to treat AP, its etiological factors should be surgically and non-surgically removed. Indications for surgical endodontics are on decrease due to their complications and adverse effects; however, they still comprise 3–10% of endodontic treatments [3]. In the modern age of endodontics, conservative/non-surgical management of endodontic pathosis is considered a priority when compared with aggressive surgical procedures [4]. To achieve successful outcomes in non-surgical approaches, regional disinfection and tissue repair are proposed [5]. Non-surgical management of periapical lesions has been reported in several investigations, where mechanical instrumentation of root canals and employment of an intracanal medicament have been suggested [6, 7]. Using intracanal medication to eliminate microorganisms and prepare an appropriate environment for further treatments seems to be a major component in non-surgical approach [8].

It has been shown that if canals are carefully cleaned and disinfected but not obturated, endodontic success could be expected [9, 10]. Consequently, intracanal medicaments have been used as adjuncts to the mechanical preparation of the root canal system. Amongst different medication, calcium hydroxide and triple antibiotic paste (TAP) have been proposed for regenerative endodontics [11]. TAP, the antibiotic combination of ciprofloxacin, metronidazole, and minocycline, has shown to remove most intracanal microorganisms and prepare a proper matrix for the regeneration and revascularisation of the pulpal tissue inside the root canal system [12]. Nonetheless, TAP has shown drawbacks mainly due to the existence of minocycline, including tooth discolouration, changes to dentine structure, and cytotoxicity. There have been various studies on the modification of TAP, and a number of replacements for minocycline, amoxicillin, arestin, and cefaclor, have been introduced to the literature [13, 14].

The objective of the current study of three hopeless cases was to report the healing of large periapical lesions through the long-term application of a new combination of triple antibiotics, Penicillin G (PG), metronidazole, and ciprofloxacin (PMC), for the disinfection of the root canal system.

## 2. Case Presentation

### 2.1. Case 1

A 33-year-old male patient was referred for the evaluation and possible treatment of previously endodontically treated tooth #18 with a large periapical lesion detected during clinical and radiographic examinations. The patient expressed severe pain in the mandibular left region and complained about a swelling in the area.

**Figure 1:**
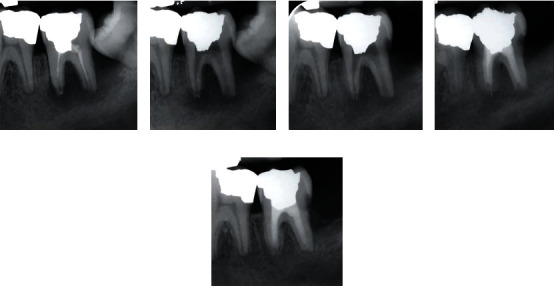
Mandibular left second molar with symptomatic apical periodontitis. (a) Pre-operative radiograph showing inadequate previous root canal treatment and a large periapical lesion. (b) After removal of the root canal obturation materials, canal preparation, and insertion of antibiotics. (c) Follow-up radiograph after 4 months and relative healing of the periapical lesion. (d) Post-operative radiograph 12 months after the initial radiograph, the obturated canals and bone healing. (e) Post-operative radiograph after 3 years from the initial radiograph, complete bone healing and thorough disappearance of the lesion.

Initially, medical and dental histories of the patient were taken. In clinical examination, tooth #18 was found to be highly sensitive to percussion, and a mobility of grade II next to swelling around the tooth was seen. Probing on the distal root showed a deep periodontal pocket, reaching the periapical area of the root. Furthermore, a mesio-angular wisdom tooth (#17) had caused food impaction and caries on the distal surface of the tooth. Periapical radiographic evaluation of the tooth #18 revealed inadequate root canal filling with a large periapical lesion and severe bone resorption around the involved tooth. The mesial/distal roots and bone in the distal aspect of the tooth seemed to have resorbed (Figure 1(a)). The tooth was diagnosed with an endo-perio lesion accompanied by symptomatic AP. Proposed treatment plans were thoroughly explained to the patient comprising (i) simple extraction of tooth #18 with/without replacement with dental implants, (ii) intentional replantation of tooth #18, (iii) simple extraction of tooth #18 with transplantation of tooth #17 as a replacement for tooth #18, (iv) endodontic retreatment of tooth #18 and apicoectomy of the roots, and (v) endodontic retreatment of tooth #18. Patient insisted on the survival of the tooth, with least possible complications. Therefore, and after detailed explanation of the possible procedures, the patient agreed on the proposed endodontic retreatment of tooth #18 and surgical extraction of the wisdom tooth. Afterwards, the informed consent was obtained.

In the first appointment, under regional local anaesthesia and rubber dam in place, necessary coronal restoration of tooth #18 was removed, an appropriate access cavity was prepared, and canal orifices were cautiously investigated, probed, and located. Using chloroform, previous root canal obturation materials were completely removed, and canals were carefully irrigated with 5.25% sodium hypochlorite (NaOCl; Morvabon Hypo Endox, Tehran, Iran). Then, the root canal system was chemomechanically cleaned, shaped, and gently/copiously flooded with normal saline. Next, the new combination of triple antibiotics [PMC; with the ratio of 1 : 1 : 1] was prepared and inserted into the root canals. Then, the tooth was temporarily dressed with Zonalin™ (i.e., re-enforced zinc oxide eugenol; Kemdent, Swindon, UK), and the patient was dismissed for regular clinical observations (Figure 1(b)). A week later, the patient showed no sign of swelling and pain. Six months later, radiographic evaluation revealed bone healing in the periapical region with mere disappearance of the radiolucent lesion. Due to the food impaction between teeth #17 and #18, the wisdom tooth was surgically removed in the same appointment. The patient was dismissed for 4 months, after which the patient reported no signs of clinical symptoms and that the tooth was not painful in mastication. In the radiographic evaluation, relative healing of the surrounding bone and disappearance of the periapical lesion were seen (Figure 1(c)). The patient was asked to recall in two months to complete the retreatment. Then, the root canals were filled with calcium-enriched mixture cement (BioniqueDent, Tehran, Iran), via mixing its liquid and powder in accordance with the manufacturer's instructions, and the tooth was coronally restored using amalgam (Cinalux Faghihi, Tehran, Iran) as the restorative material in the same session (Figure 1(d)). In 3-year follow-up, thorough healing of the bone and complete disappearance of the periapical lesion were radiographically observed (Figure 1(e)).

### 2.2. Case 2

A 35-year-old female patient with severe pain on mastication and buccal swelling on tooth #19 was referred for further consultation and/or possible endodontic treatment.

**Figure 2:**
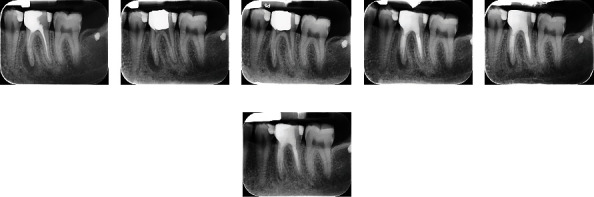
Mandibular left first molar with symptomatic apical periodontitis. (a) Pre-operative radiograph showing inadequate previous root canal treatment, broken instruments, and a large periapical lesion around the mesial root. (b) After removal of the root canal obturation materials, canal preparation, and application of intracanal medication. (c) Follow-up radiograph after 4 months and relative healing of the periapical lesion. (d) Post-operative radiograph 9 months after the initial radiograph, the bone healing and canal obturation. (e) Post-operative radiograph 17 months from the initial radiograph. (f) 28-month follow-up radiograph exhibiting complete bone healing and disappearance of the lesion.

Medical and dental histories of the patient were primarily taken, and then, clinical examination was conducted. In intraoral clinical examination, despite typical periodontal probing and normal tooth mobility, swelling on the buccal side of tooth #19 and severe pain on percussion test were seen. Initial radiographic evaluation of tooth #19 showed inadequate root canal treatment with large periapical radiolucency and massive bone resorption around the mesial root. In addition, further careful radiographic observation revealed two broken instruments in mesiobuccal and mesiolingual root canals, close to radiographic apices. Moreover, inadequate root canal obturation was seen in the mesial and distal canals, with a small radiographic lesion surrounding the distal root (Figure 2(a)). The tooth was diagnosed with symptomatic AP. Possible treatment options were thoroughly explained to the patient comprising: (i) simple extraction of tooth #19 with/without replacement with dental implants, (ii) intentional replantation of tooth #19, (iii) endodontic retreatment of tooth #19 and apicoectomy of the roots, and (iv) endodontic retreatment of tooth #19. The patient wished to keep her tooth with minimum possible complications; consequently, the endodontic retreatment of tooth #19 was thoroughly re-explained to the patient, and her informed consent was formally obtained.

In the first appointment, after tooth isolation with rubber dam, anesthesia, removal of the tooth previous restorative materials, and localization of canal orifices, the obturation materials were totally removed from the canals, following the same treatment protocol in Case 1. Subsequently, the radicular canals were cleaned, shaped, and completely prepared with copious amounts of normal saline. In the mesial canals, and due to the blockage made by broken instruments left from the previous treatment, canals were carefully prepared and entirely irrigated to the place of broken instruments. Then, PMC was prepared (same formulation as Case 1) and applied into the canals for the disinfection and removal of microorganisms. Next, the tooth was permanently restored with dental amalgam (Cinalux Faghihi), and the patient was dismissed for regular postoperative sessions (Figure 2(b)). After a week, the patient reported no sign of drastic pain and decrease in the swelling. After 4 months, radiographic evaluation showed initial healing of the bone surrounding the mesial and distal roots. Therefore, the restoration was removed, the canals were irrigated, PMC was re-applied into the canals, the tooth was restored, and the patient was dismissed for the next appointment (Figure 2(c)). In 9-month follow-up, radiographic evaluation showed large decrease in the size of the mesial and distal radiolucent lesions, and there was no sign of pain or swelling on/around the tooth. In consequence, the canals were completely irrigated, refreshed (re-prepared), and obturated with gutta-percha (BioMed, Chungcheongbuk-do, South Korea) using lateral condensation technique. The tooth was restored with amalgam (Cinalux Faghihi), and the patient was dismissed for another follow-up session (Figure 2(d)). In a 16-month follow-up, complete healing of the bone around the distal root was observed, whereas the periapical lesion around the mesial root had become much smaller in comparison with the initial diagnostic radiograph (Figure 2(e)). In 28-month follow-up, radiographic evaluation showed thorough healing of the bone around the distal root and small radiolucency around the mesial root. The patient reported no complaint regarding swelling and pain on percussion (Figure 2(f)).

### 2.3. Case 3

A 30-year-old female patient with a compromised previous endodontic treatment of tooth #30 was referred for further consultation and necessary endodontic treatment. The patient complained about localized swelling, severe pain, and extreme discomfort during mastication on tooth #30.

**Figure 3:**
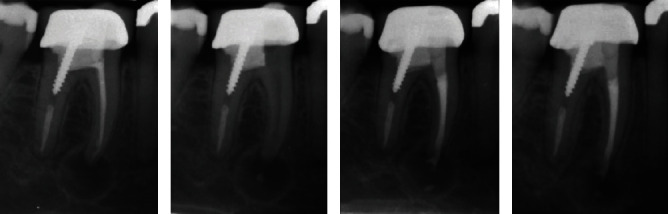
A mandibular right first molar with symptomatic apical periodontitis. (a) Diagnostic periapical radiography showing compromised previous endodontic treatment and a large periapical radiolucency around the mesial root. (b) In-treatment periapical radiography demonstrating conservative preparation of access cavity, removal of obturation materials, and proper cleaning and shaping of the mesial canals. (c) Immediate post-treatment radiography, revealing periapical lesion regression with clinical disappearance of pain and relative healing of the regional swelling. (d) Post-operative radiograph after 10 months of the initial treatment, exhibiting bone healing and progressive disappearance of the large periapical radiolucency.

Patient's medical and dental histories were primarily obtained, and clinical examination was then conducted. In the intraoral clinical examination, tooth #30 showed no abnormal mobility; however, a localized large swelling was seen on the buccal side of tooth #30 with severe pain reported on percussion. Radiographic evaluation revealed an inappropriate previous endodontic treatment in the mesial canals as well as a large periapical radiolucency surrounding the mesial-root periapical region with bone resorption evident in the area; nevertheless, the endodontic treatment conducted in the distal root, post insertion, and the crown restoration seemed to be satisfactory (Figure 3(a)). The possible treatment plans, explained thoroughly to the patient, included: (i) simple extraction of tooth #30 with/without replacement with dental implants, (ii) intentional replantation of tooth #30, (iii) endodontic retreatment of tooth #30 and apicoectomy of the mesial root, (iv) bi-cuspidisation, and (v) endodontic retreatment of tooth #30. The patient agreed on the most conservative approach with minimal possible adverse effects; i.e. the endodontic retreatment of the tooth. Therefore, her informed consent was obtained.

In the first treatment session, after the administration of local anaesthesia and rubber-dam isolation of tooth #30, a conservative access cavity was prepared through the coronal restoration. Then, the obturation materials were cautiously removed from the pulp chamber (the same protocol as Case 1), and mesial canals were thoroughly cleaned and shaped. Next, PMC was prepared (same formulation as Case 1) and transferred to the mesial canals, and tooth #30 was temporised with Zonalin™ (Kemdent; Figure 3(b)). After 2 weeks, the patient expressed no pain on mastication, the swelling had regressed substantially, and radiographic re-evaluation showed relative bone healing around the mesial root. Then, the dressing was removed, and the mesial canals were irrigated with copious amount of normal saline. Subsequently, the canals were dried, refreshed, and obturated with gutta-percha (BioMed) using lateral condensation technique. Successively, the tooth was restored permanently with resin-based dental composite restorative material (3M™ Filtek™ Z250, USA), and the patient was dismissed (Figure 3(c)). After 6 months of the final restoration and 10 months of the initial treatment, the tooth remained completely functional and asymptomatic with perfect bone healing and considerable disappearance of the periapical lesions (Figure 3(d)).

## 3. Discussion

In the present case series study, PMC was used as the intracanal medicament. TAP, despite its advantages, has shown disadvantages, principally due to the presence of minocycline [12]. PG has white appearance similar to that of ciprofloxacin and metronidazole [15], and with the two antibiotics, can make a strong antibacterial combination in a white-coloured powder. However, in vitro and clinical observations are necessary to confirm the hypothesis. Furthermore, PG is a broad spectrum antibiotic and can effectively act against Gram positive bacteria, although there are reports that PG can be an effective antibiotic against Gram negative microorganisms [16]. One important advantage of PG over minocycline is that PG is effective against *Enterococcus faecalis* (*E. faecalis*) [17], whereas minocycline has shown inability to act against the microorganism [18]. *E. faecalis* seems to be the most dominant microorganism in endodontic failures [19]. Therefore, PMC can act against the bacterium and play an important role in the combat/treatment against endodontic failures. The favourable outcomes described in the three cases treated with PMC showed that the newly/recently introduced combination of triple antibiotics, when accompanied by the mechanical preparation of root canals without prompt obturation, could lead to endodontic success in previously unsuccessful/compromised endodontically treated teeth with AP and large periapical lesion. PMC has recently been loaded on an innovative local drug delivery system [17] to be transferred to the radicular area, achieve target drug delivery, and combat intracanal microbial species [20, 21].

The postoperative radiographs of the reported cases showed regression of large periapical lesions, disappearance of mobility (Case 1), and thorough healing of bone. It seemed that PMC could (a) function well against intracanal and periapical microorganisms, (b) result in the removal of bacteria and disinfection of the region, and (c) end in the regeneration of bone tissue and eventually healing of the large periapical lesion surrounding the roots in the course of treatment. Therefore, throughout the ministration, the intracanal dressing managed to combat the intracanal pathogenic microbiota, remove the causative microorganisms, as well as preparing an environment/proper grounds for subsequent healing of the lesions. These findings were similar to the outcomes of an investigation conducted by Kusgoz et al. [22], where the TAP caused the disappearance of periapical lesions and healing of the surrounding bone. In another comparable study, Holland et al. [23] showed the importance of intracanal medicaments in the healing of large periapical lesions.

In the current study, the ministered teeth were completely functional after the treatment sessions although the root canals were not initially obturated, and no signs of swelling/pain were reported by the patient. The large periapical lesions were gradually replaced by newly formed bone, and peri-radicular healing seemed complete. The outcome of our cases was consistent with the treatment protocols introduced by Asgary and Fazlyab [10] and Shah and Logani [24], who endodontically treated the involved teeth with proper canal preparation and intracanal antibiotics without immediate obturation.

The current case reports presented a follow-up period of 36, 28, and 10 months for cases 1, 2, and 3, respectively. The length of follow-up could differ in various studies; Pieper and Piva [25] conducted their research on the regression of a large periapical lesion using nonsurgical endodontic treatment over a period of two months, whereas Kusgoz et al. [22] chose 30 months to evaluate his final outcomes. The effectiveness of the treatments is better evaluated with longer follow-ups [26].

Besides, it appeared that eventual complete obturation of the root canals managed to favour the repair in the periapical and peri-radicular tissues. This result followed a similar concept presented by Holland et al. [27], who showed the significance of root canal obturation in the repair of the surrounding regions. Moreover, this concept is in line with the ideas expressed by Shah [28] and Jha et al. [29], whose studies exhibited the importance of biomechanical preparation of the radicular system and the application of an intracanal medication in achieving successful outcomes of endodontic treatments.

## 4. Conclusions

Using PMC, as a newly/recently introduced intracanal medication, to disinfect root canal system with proper cleaning and shaping of radicular canals, seems to successfully manage large periapical lesions and result in desirable endodontic outcome(s). It caused hopeless teeth to re-function normally despite initial poor prognoses. Moreover, the mentioned minimally invasive method was cost-effective and required a relatively simple clinical approach. Nevertheless, additional evaluation of the consequences and further well-designed clinical trials are recommended.

## Data Availability

The data used to support the findings of the current study are available from the corresponding author upon reasonable request.
